# The Characteristics of Human Genes: Analysis of Human Chromosome 22

**DOI:** 10.1002/cfg.335

**Published:** 2003-12

**Authors:** Ian Dunham, David M. Beare, John E. Collins

**Affiliations:** The Wellcome Trust Sanger Institute Wellcome Trust Genome Campus Hinxton, Cambridge CB10 1SA UK


**Comparative and Functional Genomics**
*Comp Funct Genom* 2003; **4**: 635–646.
Published online in Wiley InterScience (www.interscience.wiley.com). **DOI:** 10.1002/cfg.335

**Conference Review**

### The characteristics of human genes: analysis of human chromosome 22

Ian Dunham*, David M. Beare and John E. Collins
*The Wellcome Trust Sanger Institute, Wellcome Trust Genome Campus, Hinxton, Cambridge CB10 1SA, UK*

**Correspondence to:*
*Ian Dunham, The Wellcome Trust Sanger Institute, Wellcome Trust Genome Campus, Hinxton, Cambridge CB10 1SA, UK.*
*E-mail: id1@sanger.ac.uk*

Received: 15 August 2003
Revised: 4 September 2003
Accepted: 8 September 2003

### Introduction

On 14 April 2003 *Homo sapiens* became the first species on earth to finish reading its own set of instructions. However, although we have read our code, the problem that now faces us is to understand what we have read. The first task towards this understanding is to establish the full catalogue of human genes based on the genome sequence. Although evidence is accumulating that questions our assumptions about what constitutes a gene and it seems possible that we may have to broaden our horizons to include various classes of non-protein coding RNAs, a first generation gene catalogue must inevitably be focused on protein-coding genes.

This layering of information onto sequence is widely called 'annotation' and we term the specific process of describing gene structures on the genomic sequence 'gene annotation'. Although ultimately the genome sequence should be accompanied by an information-rich annotation describing many aspects of structure and function, for the moment accurately describing gene structures remains a considerable task. Despite progress in methods for gene prediction, and the availability of genome sequence from other mammals for comparative analysis, deriving the gene catalogue by computation is still an imperfect art (Guigo *et al.*, 2000). On the other hand, curation of gene structures supported by experimental data, either from the cDNA and EST databases, or more unusually from *de novo* cloning and sequencing, is labour-intensive and has not yet been applied to the full genome. A particular problem is that most cDNA libraries are derived from total cellular RNA and contain a high proportion of unprocessed and partially processed RNA species, confounding the identification of intron–exon junctions from ESTs or cDNA sequences (Bashiardes and Lovett, 2000). Thus, the protein coding gene catalogue is far from complete. What we have instead are a series of attempts to approximate or estimate what the gene catalogue looks like, based on applying the current favourite gene finding paradigms to the available genome sequence (International Human Genome Sequencing Consortium, 2001; Das *et al.*, 2001; Davuluri *et al.*, 2001; Ewing and Green, 2000; Flicek *et al.*, 2003; Guigo *et al.*, 2003; Liang *et al.*, 2000; Roest Crollius *et al.*, 2000; Shoemaker *et al.*, 2001; Wright *et al.*, 2001).

We have taken an alternative approach by concentrating on a single contiguous segment, representing 1% of the human genome, and attempting to produce a highly curated gene annotation, supported by expressed sequences from the databases and experimental confirmation of gene structures

Copyright © 2003 John Wiley & Sons, Ltd.

by amplification and sequencing from cDNA in the laboratory (Collins *et al.*, 2003; Dunham *et al.*, 1999). As far as possible we have established canonical gene structures which include the 5′ and 3′ ends for every gene, giving a single comprehensive gene set. Our motivation for this approach was that, in addition to producing a highly curated dataset which could be used as a benchmark for testing novel approaches to gene finding, extrapolation from this 1% could lead to insights about the full human gene set. The region that we chose to study, for largely historical reasons, was the long arm of human chromosome 22. This choice can be criticized on the basis that 22q is a somewhat exceptional region of the genome. It is unusually GC-rich, *Alu*-rich, LINE-poor and replicates very early in the cell cycle compared with other human chromosomes. However, although these factors make chromosome 22 exceptional as a chromosome, they also make 22q the typical environment of the vast majority of protein-coding genes. Thus, we believe that there is some value in describing the overall properties of the chromosome 22 genes as an indication of the qualities of the other human genes.

### Overall properties of the chromosome 22 gene annotation

We have recently published an extensive reanalysis of the gene content of human chromosome 22 (Collins *et al.*, 2003). In this gene annotation, we aimed to identify genes, and their canonical genomic structures, supported by evidence from transcribed sequences across the entire gene length. After subjecting the genomic sequence of chromosome 22 to a suite of bioinformatic analyses, we aligned full-length cDNA or assembled EST to the genome and resolved splice sites and 3′ ends. We then extended incomplete genes, joined 5′ and 3′ EST clusters, or confirmed preliminary gene structures by obtaining additional cDNA sequence using directed cDNA or reverse transcriptase (RT)–PCR and sequencing. All the fragments sequenced were realigned to the chromosome 22 sequence and the gene structures were updated. Having established the annotation, open reading frames (ORFs) of greater than 300 bases were identified and we categorized the genes based on their structural features. Essentially, genes with support from transcribed sequences with identifiable ORFs were split into either complete or partial protein-coding genes depending on whether a 5′ and 3′ end had been confirmed according to defined criteria. Other structures supported by transcribed sequences but without ORFs were classified as non-coding RNAs. In addition, we annotated a series of processed and duplicated pseudogenes and postulated that some of the partial genes, while still containing ORFs and so not meeting our definition of a pseudogene, may represent 'prepseudogenes'. Table 1 of Collins *et al.*

**Table 1.** Basic properties of genes on human chromosome 22

| | All structures | Genes with ORF | ORFs | Pseudogenes |
|---|---|---|---|---|
| Number of annotations | 936 | 461 | 461 | 234 |
| Total genomic coverage | 18.6 Mb (55%) | 17.0 Mb (50.3%) | 13.9 Mb (41.1%) | 0.84 Mb (2.5%) |
| Mean gene size | 19921 | 36960 | 30190 | 3580 |
| Median gene size | 3730 | 16451 | 12319 | 1468 |
| Mean exons per gene | 5.5 | 9.1 | 8.4 | 2.2 |
| Median exons per gene | 3 | 7 | 6 | 1 |
| Mean exon size | 350 | 317 | 171 | 685 |
| Median exon size | 139 | 134 | 122 | 216 |
| Mean cDNA length | 1935 | 2889 | 1454 | 1511 |
| Median cDNA length | 1392 | 2298 | 1104 | 1173 |
| Total exon length | 1.81 Mb (5.2%) | 1.3 Mb (3.7%) | 0.7 Mb (2%) | 0.35 Mb (1%) |
| Total intron length | 16.8 Mb | 15.7 Mb | 13.2 Mb | 0.49 Mb |
| % removed as intron | 90% | 92% | 95% | NA |

'All structures' refers to the complete annotation, including the gene segments of the immunoglobulin-λ cluster and pseudogenes. 'Genes with ORF' includes only gene structures with an annotated ORF of 300 bases or greater, but not immunoglobulin-λ segments. Only 387 of these genes were defined as full-length. 'ORFs' refers to only the annotated ORF structures. Genomic coverage is calculated on the basis of the fraction of the known sequence covered, excluding sequence gaps (33 821 705 bp). Unless otherwise indicated, sizes are in bases.

Copyright © 2003 John Wiley & Sons, Ltd. 

(2003) summarizes the distribution of gene structures between these categories and is the starting point for the additional characterizations we report here. The complete annotation set and the associated reference sequence are archived and available at **http://www.sanger.ac.uk/HGP/Chr22**.

Table 1 shows the summary characteristics of chromosome 22 genes from the annotation described in Collins *et al.* (2003). Overall, 55% of the 33.8 Mb of 22q genomic sequence is covered by gene structures, including non-protein-coding genes and pseudogenes. If we ignore pseudogenes, non-coding RNAs and the gene segments of the immunoglobulin-λ gene cluster, then classical protein-coding genes cover 51% of 22q. Only 3.7% of the 22q sequence is protein coding gene exon, and a mere 2% of the sequence actually codes for amino acids. Looking at this in another way, while at least 51% of the sequence is transcribed, 92% of the transcribed sequence is removed by processing before mature message is produced. Pseudogenes are an order of magnitude smaller than protein-coding genes, but are numerous and cover 2.5% of 22q, or 1% if intronic sequences are ignored. Thus, the amounts of the chromosome given over to protein coding exons and pseudogenes are surprisingly similar. For this paper, we will concentrate on the properties of the protein-coding genes on chromosome 22.

### The structure of protein-coding genes

### Gene size and exon number

A typical protein coding gene is about 36.9 kb in length and contains 9 exons. However, these figures mask a distribution with a long tail containing a small number of genes, which are either genomically large, or have many exons, or both. Figure 1 illustrates this distribution for the genomic span of chromosome 22 genes. The largest gene, cB42E1.1, is 701 kb in length and is followed by genes spanning 647 kb (LARGE) and 544 kb (SYN3). The genes with the largest genomic expanse are not the ones with the most exons, these being instead PICK4CA with 55 exons across 150 kb and MYO18B with 44 exons spanning 288 kb. However, there is a significant general relationship between gene size and exon number with larger genes having more exons [Figure 1C; Spearman correlation coefficient $(r) = 0.6844$ at $p < 0.0001$]. At the other end of the spectrum, 19 genes which meet our criteria for full-length protein-coding genes consist of only a single exon, with some smaller than 2 kb in length.

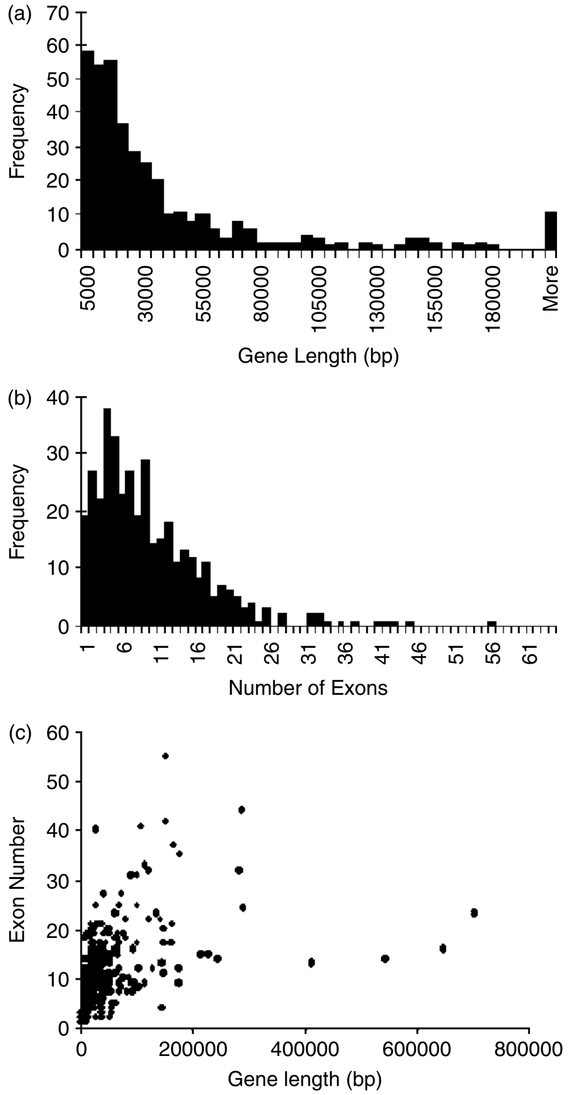

**Figure 1.** Distributions of exon number and gene size for 387 chromosome 22 full-length protein-coding genes

### Genomic locations and GC content

It has long been known that genomically large genes tend to reside in GC-poor, *Alu*-poor and LINE-rich regions of the genome, whereas small genes reside in genomic regions with the opposite characteristics (Duret *et al.*, 1995). This is confirmed for the chromosome 22 protein coding gene

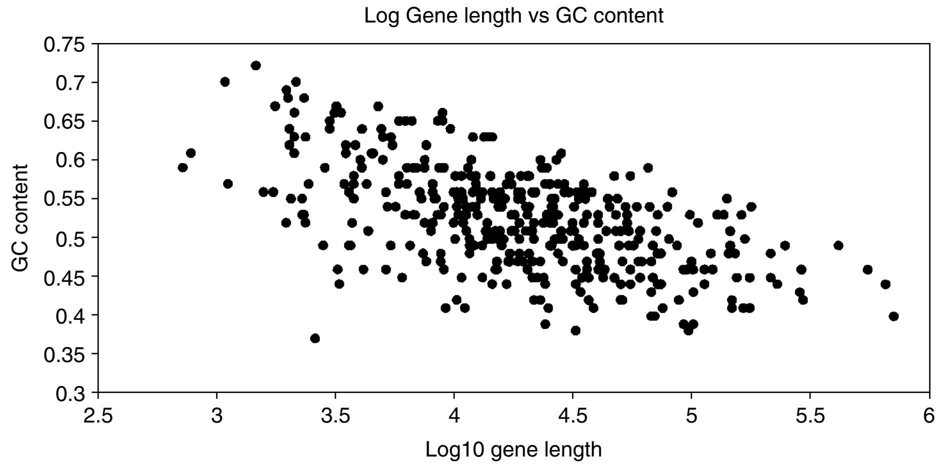

**Figure 2.** The relationship of gene length to GC content. GC content was calculated for the genomic span of each of the 387 full-length protein-coding genes and plotted against $\log_{10}$ of the gene length. Pearson's correlation coefficient was determined as −0.5728 at $p < 0.0001$

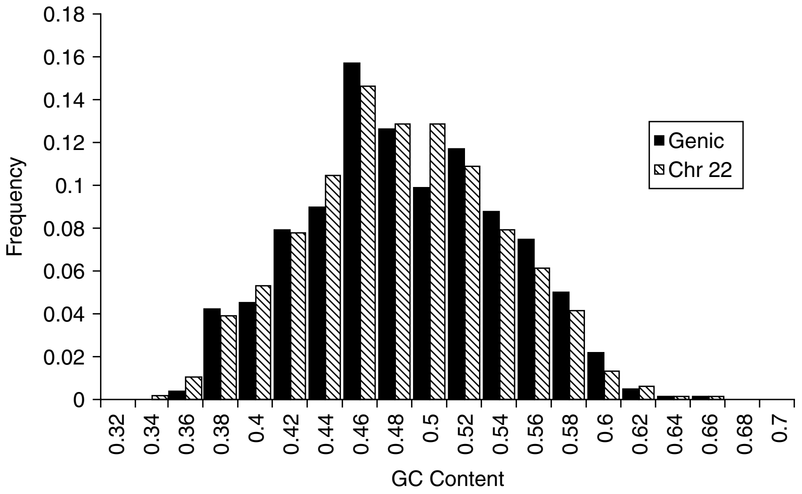

**Figure 3.** GC content distributions for genes and total chromosome 22 DNA. The whole chromosome 22 sequence and the intragenic sequence for the 387 full-length protein-coding genes only were analysed for GC content in non-overlapping 20 kb windows. The distributions were then adjusted so that the frequencies summed to 1

set in Figure 2 which shows the inverse correlation between GC content of the genomic span of the gene and $\log_{10}$ of gene length [correlation coefficient $(r) = -0.5728$ at $p < 0.0001$]. As has previously been pointed out, this effect is due to increased intron lengths in GC-poor regions (International Human Genome Sequencing Consortium, 2001), and exon number is only weakly inversely correlated with GC content (data not shown). However, contrary to expectations from the whole genome, there is little difference in the patterns of GC content between the regions of chromosome 22 that contain genes (intragenic) and those that do not (intergenic). Figure 3 shows the distribution of GC contents for the genomic spans of the chromosome 22 protein-coding genes in 20 kb non-overlapping windows compared to the overall GC content of chromosome 22. The distribution of GC content for chromosome 22 sequence as a whole is remarkably similar to that for the genes alone (as is the distribution for intergenic DNA alone; data not shown) which is unlike the equivalent

Copyright © 2003 John Wiley & Sons, Ltd. 

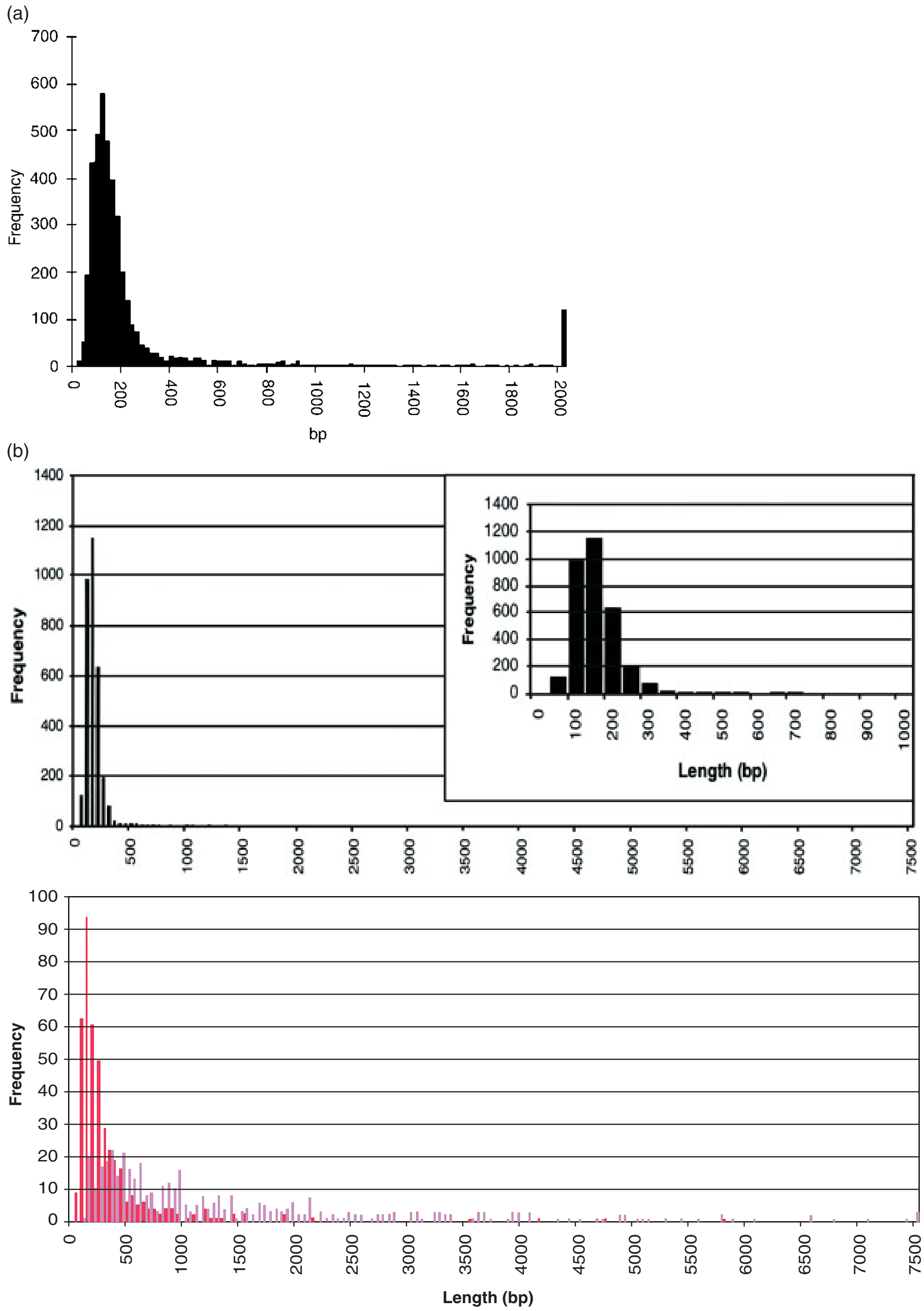

**Figure 4.** Distributions of exon sizes for 461 chromosome 22 genes with ORFs. (A) Frequency distribution for all chromosome 22 protein-coding exons by length. The combined frequencies for all exons greater than 2000 bp in size are grouped in the frequency bar at the right of the distribution. (B) Frequency distribution for all chromosome 22 protein-coding exons by length, broken down by exon type. The top panel shows the frequency distribution for internal exons (black bars), with the inset showing a blow-up of the class intervals up to 1 kb. The bottom panel shows the same distribution for 5′ (black) and 3′ (grey) exons. Length class interval labels refer to the upper limit of the interval, i.e. 50 represents the interval 1–50 bases, etc.

Copyright © 2003 John Wiley & Sons, Ltd. 

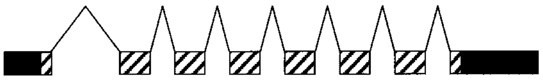

| | 5' exon | Internal exons | 3'Exon |
|---|---|---|---|
| Mean size | 321 bp | 150 bp | 1433 bp |
| Median Size | 185 bp | 121 bp | 857 bp |
| Largest | 5792 bp | 5209 bp | 12954 bp |
| Smallest | ND | 6 | ND |

**Figure 5.** Summary statistics for different exon types for the 461 protein-coding genes. The diagram at the top represents a prototypical exon/intron structure for a chromosome 22 gene, with the boxes representing the exons, and the hashed regions representing the most common exon organization of the ORF. Statistics for each type of exon are given underneath. Smallest exon sizes for 5′ and 3′ exons are not given, as we cannot be sure we have annotated to the full extremity of these exons in some cases distributions for the whole genome (see Figure 36 of International Human Genome Sequencing Consortium, 2001). Thus both intra- and intergenic GC content are shifted towards a higher GC-content on chromosome 22 than is seen for the whole genome. This difference appears to be because chromosome 22 does not contain the large, GC-poor gene deserts that are found elsewhere in the genome. In fact several substantial regions of 22q13 contain no annotated genes but are extremely GC-rich.

### Exon and intron structure

Looking in more detail at the structure of the protein-coding genes, a typical gene codes for a cDNA of 2–3 kb in 7–9 exons. The mean and median exon sizes are given in Table 1 and the distribution of exon sizes for all exons is shown in Figure 4A. However, examining exon sizes grouped by type of exon presents a more accurate picture, since 5′ exons and particularly 3′ exons are both generally larger than internal exons, and have a more varied distribution (Figure 4B). Figure 5 summarizes the descriptive statistics for each type of exon. As was seen previously (International Human Genome Sequencing Consortium, 2001) it is notable that most internal exons are tightly grouped around the 101–150 bp interval, with only a handful of very short exons, the very smallest of which have a purine bias (61.5% purine, 38.5% pyrimidine for exons of <19 bp). The smallest internal exon is 6 bp in bK57G9.2. Some large internal exons do occur, with the largest being 5209 bp. Of more interest is that with this chromosome 22 dataset, where we have carefully defined the extent of the 5′ and 3′ exons, we are able to give more accurate statistics for these exons than has previously been possible. For the 5′ exons, the distribution peaks around the 101–150 bp interval and has a median of 185 bp. However, there is a much longer tail than seen with internal exons, reflecting the general tendency for 5′ exons to be larger and more variable in length than internal exons. Looking at 3′ exons, the distribution is much flatter and ranges up to much larger exons, giving a median of 857 bp. The largest 3′ exon is 12.9 kb, in the dJ1042K10.4 mRNA. Thus, 3′ exons are apparently unconstrained in size, unlike internal exons, presumably due to independence from the restrictions of the splicing machinery.

Ten pairs of protein coding gene structures overlap with each other, half orientated head-to-head, and half tail-to-tail. A further 21 pairs of the full-length protein-coding genes (10.7%) are orientated head-to-head, with transcriptional starts within 2 kb of each other. In all cases, a single CpG island is located between the genes, which might act as the promoter. These are candidates to be under the control of a bidirectional promoter.

The introns of genes are generally regarded as of little interest. However, on chromosome 22 we identified 11 protein-coding genes which lie within a single intron of another gene on the opposite strand. Four of these 'genes within genes', TIMP3, SERPIND1, GNAZ and U51561.2, are complete. The remaining seven are partial genes, of which three are spliced. A further three snoRNA genes lie in the same transcriptional orientation within introns of the RPL3 gene. There is also a length bias observed for the first intron of full-length

Copyright © 2003 John Wiley & Sons, Ltd. 

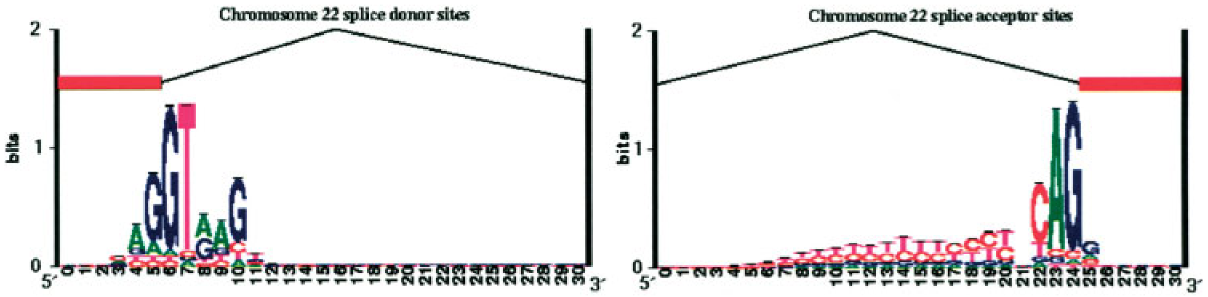

**Figure 6.** Sequence Logo (Schneider and Stephens, 1990) showing the splice donor and acceptor consensus sites in 3857 chromosome 22 confirmed introns genes. A comparison of the lengths of the first intron of complete genes with more than two exons to the mean lengths of the remaining introns showed that the difference is significant and the first intron is on average 1.8 times larger (comparing medians, Wilcoxon matched-pairs signed-ranks test $p < 0.0001$). This phenomenon means that the transcriptional start and the first exon of a gene can often be at considerable distance from the remaining coding exons. It has previously been speculated that this size difference could be consistent with transcriptional control features being present in the first introns (Chen *et al*., 2002), although it could also be consistent with less constraints on intron size for the first intron, given that some can be many tens of kilobases in size.

### Splice sites

Analysis of 3857 introns from all the chromosome 22 annotations except pseudogenes shows a high similarity to the splice donor and acceptor consensus motifs defined from a study of ∼1800 introns by Stephens and Schneider (1992) (Figure 6). The only variations are bases 18–20 of the splice acceptor where thymidine is most frequent in Stephens and Schneider, but in this analysis cytosine is more common. Only three out of 3857 acceptor sites are not preceded by an intron ending with AG, instead they have AC, TC or GT. At the splice donor site, 23 introns do not begin with the canonical GT, instead beginning with GC 15 times, AT three times, GA twice, and TA, CT and CC once each. The gene CACNA1I not only contains the only U12-type AT-AC intron in this set (Mittman *et al*., 1999), but also two GC splice donor sites, making it the only gene to have more than one variation from the splice consensus.

### 5′ Ends and promoters

*In silico* prediction of candidate promoter sequences or transcriptional starts has been the subject of considerable effort recently (Davuluri *et al*., 2001; Down and Hubbard, 2002; Scherf *et al*., 2000). We examined our set of 391 annotations that had been assigned a probable 5′ end and ascertained the success of a series of promoter or

**Table 2.** Results of promoter prediction programs compared to 391 chromosome 22 genes with probable 5′ ends

| Method | Total predictions | True promoters matched (sensitivity) | Proportion of true promoter matches (specificity) |
|---|---|---|---|
| CpG Islands | 546 | 324/391 (83%) | 331/546 (61%) |
| Eponine | 2055 | 274/391 (70%) | 1313/2055 (64%) |
| Genomatix | 418 | 280/391 (72%) | 279/418 (67%) |
| First EF | 1447 | 227/391 (58%) | 637/1447 (44%) |
| All | 4466 | 361/391 (92%) | 2560/4466 (57%) |

The genomic sequence was searched with CPGFIND (Micklem, unpublished), which predicted a CpG island if the GC content was greater than 60%, the ratio of observed CpG frequency/expected CpG frequency was greater than 0.6 and there were more than 200 bases of CpG island DNA. Eponine was downloaded from **http://www.sanger.ac.uk/Users/td2/eponine** and was run on the sequence with the threshold set at 0.999. Analyses for PromoterInspector (Scherf *et al*., 2000) and First-EF (Davuluri *et al*., 2001) were obtained from the websites as described in the publications. Promoter predictions were then compared against annotated 5′ ends, and declared a match if they were within 1 kb. The results are expressed as either the number of true promoters matched out of the target set of 391 promoters (i.e. the sensitivity) or as the number of predictions matching to the target set of promoters out of the total number of predictions (i.e. the specificity). Calculations are on a per-gene basis, so that where two genes are close in head-to-head orientation, identifying this start is counted twice.

Copyright © 2003 John Wiley & Sons, Ltd. 

transcriptional start prediction programs in identifying the sequences directly overlapping or 5′ of these annotations (Table 2). Surprisingly, the best results, in terms of sensitivity to detect real promoters/gene starts, are obtained with the oldest method, simply detecting CpG islands (CPGFIND). The specificity of this method is slightly lower than the others, but only fractionally. Thirty-seven of the 67 gene starts which were not detected by CPGFIND were detected by Genomatix, Eponine or First EF, so that only 30 (7.6%) of the probable 5′ ends were not predicted. First EF made the major contribution to the additional 37 promoters identified, which were not detected by CPGFIND. Based on this experience, and given that at least 60% of human genes have CpG islands, finding CpG islands should always be the first approach for prediction of promoters. However, a small but significant gain can be obtained using multiple prediction programs. It should also be noted that the specificity of all the methods is not optimal and, although some of this is due to our exclusion of partial genes in this analysis, at least 30% false positives can be expected. Since chromosome 22 is particularly GC-rich, this gene set may be biased towards genes with CpG islands and the other prediction programs may be more valuable in other genomic regions.

### 3′ Ends and polyadenylation sites

A total of 447 chromosome 22 annotations had a confirmed 3′ end, based on the presence of a polyA tract in an exact match expressed sequence which was not found in the genomic sequence. We analysed the final 60 bases of each of these sequences for the presence of putative polyA addition signal hexamers. The most common hexamer, AATAAA (Beaudoing *et al*., 2000), accounted for 303 (68%) annotations. The mean position of the first base in the hexamer was 25 bases from the 3′ end (SD = 7). ATTAAA was present in 66 (15% of the total) of the remaining 3′ ends. One base variants from the AATAAA motif were identified in 60 (13% of the total) of the 78 annotations which did not contain either AATAAA or ATTAAA. The final 18 (4%) annotations contained a hexamer with

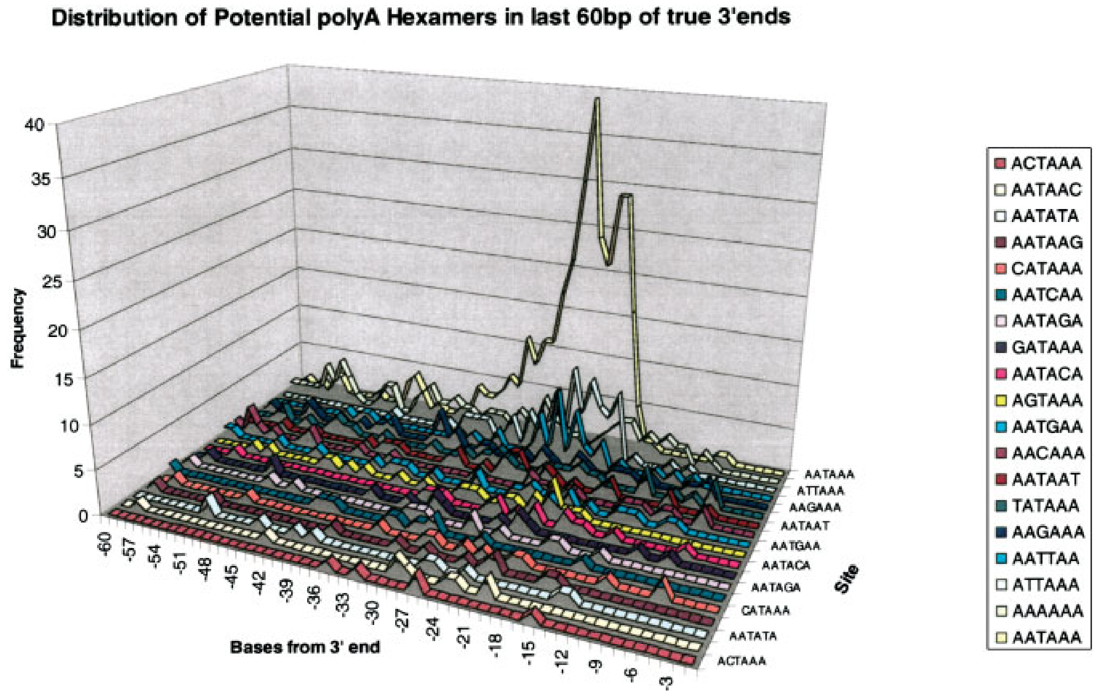

**Figure 7.** The distributions and positions of all possible polyA addition hexamers in the set of 447 confirmed 3′ ends. Frequencies for each of the hexamers in the last 60 bases of each transcript are shown. Positions are plotted at the most 5′ base of the hexamer, with coordinates measured in the 5′–3′ orientation from the transcript terminus, setting the last base of the transcript to be base −1

Copyright © 2003 John Wiley & Sons, Ltd. 

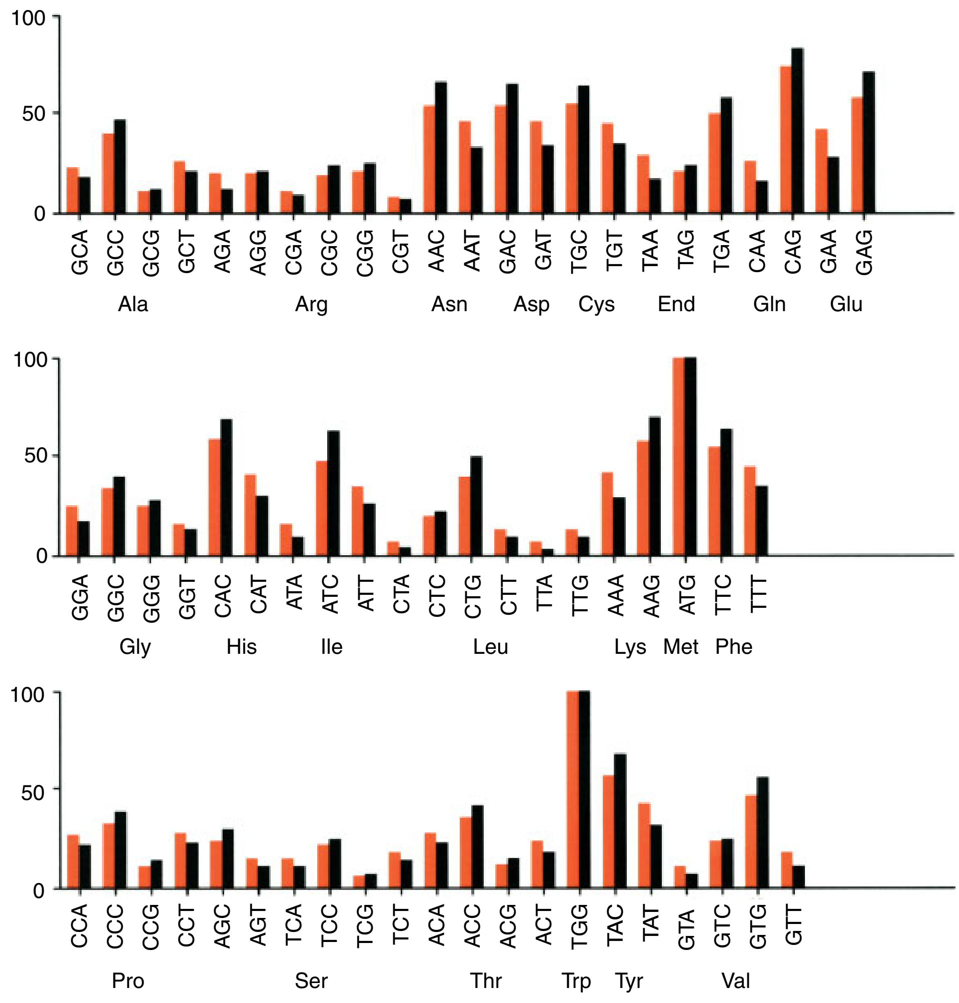

**Figure 8.** Comparison of codon usage on chromosome 22 with all human genes. Bars show the usage for each codon expressed as a percentage of all available codons for that amino acid. The human gene codon usage table at **http://www.kazusa.or.jp/codon/cgi-bin/showcodon.cgi?species=Homo+sapiens+[gbpri]** (grey bars) was compared to the codon usage data from 387 complete ORFs from chromosome 22 (black bars), using the software tool gcua at **http://gcua.schoedl.de/seqoverallex.html** (Markus Fuhrmann, Lars Ferbitz, Amparo Hausherr, Thomas Schödl and Peter Hegemann. Monitoring expression of nuclear genes in *Chlamydomonas reinhardtii* by using a synthetic luciferase reporter gene. Manuscript in preparation, 2003). The mean difference in codon usage between the two sets was 6.6%

two variations from AATAAA. Figure 7 shows the distributions and positions of all possible polyA addition hexamers in the set of confirmed 3′ ends.

### Open reading frames

The mean size of all ORFs annotated on chromosome 22 is 1454 bases. However, this includes annotations that are not full-length. There are 387 annotations for which full-length ORFs can be defined, with a mean ORF length of 1531 bases. The codon usage table for this set of ORFs is available at **http://www.sanger.ac.uk/HGP/Chr22/c22codonusage.html**. Figure 8 shows that codon usage for the subset of genes on chromosome 22 is similar to that for a large set of human genes, but is biased towards use of G or C bases rather than A or T in the third codon position. It is likely that this reflects the GC-rich nature of chromosome 22 as GC content in the third codon position (GC3) has previously been shown to correlate with GC content of the gene environment (Clay *et al.*,

Copyright © 2003 John Wiley & Sons, Ltd.

1996). The mean GC3 for the chromosome 22 genes is 69.4% and GC3 is indeed highly correlated with the GC content of the genomic segment containing the gene [Spearman correlation coefficient $(r) = 0.6891$, $p < 0.0001$].

In addition to the conventional genetic code, the chromosome 22 gene set also contains two genes that incorporate selenocysteine (U) as an alternative to terminating at a TGA codon. These are AC005005.7 and TR. Altogether, at the time of writing, there are 43 human cDNA entries in the EMBL database that indicate use of selenocysteine codons, so the frequency on chromosome 22 does not appear excessive.

Kozak has proposed the scanning model for initiation of translation, where a 40S ribosome subunit/factor complex binds to the 5′ end of the transcript and migrates to the first ATG with a strong or adequate consensus (Kozak, 1999). We

**Table 3.** Analysis of Kozak consensus sequences for 391 ORFs

| Kozak type | Site | Number | % |
|---|---|---|---|
| Strongest | GCCACCATGG | 2 | 0.5 |
| Strong | AnnATGn | 143 | 36.6 |
| | GnnATGG | 96 | 24.6 |
| | Strong total | 241 | 61.6 |
| Adequate | GnnATGY | 52 | 13.3 |
| | YnnATGG | 36 | 9.2 |
| | Adequate total | 88 | 22.5 |
| Others | GnnATGA | 25 | 6.4 |
| | None found | 37 | 9.4 |
| | Others Total | 62 | 15.9 |

annotated ORFs based on the longest single ORF found in the gene structure, independent of the presence or absence of a Kozak site. Therefore, we examined 391 annotations with an annotated ORF and a 5′ untranslated region greater than three bases for the presence of a Kozak consensus motif (Table 3). Two complete genes, NPTXR and MN1, which have a CTP start codon rather than an ATG start, were not included in this analysis. 241 (61.6%) of these ORFs conformed to the Kozak strong consensus (AnnATGN or GnnATGG), 88 (22.5%) had an adequate consensus (GnnATGY or YnnATGG) and 62 (15.9%) did not conform to the Kozak consensus sequence. Further analysis of the annotations without a Kozak consensus ATG revealed 25 with a GnnATGA motif. These included a number of well-studied genes and therefore we proposed that this is an alternative adequate initiation site. A compositional analysis of these sites is shown in Figure 9. For 32 of the remaining 37 ORFs an alternative start codon with a strong, adequate or GnnATGG consensus was found downstream in the same coding frame as the annotated longest ORF. In these cases, use of the alternative downstream start would lead to a mean reduction in size of the expressed peptide by 56 amino acid residues. In the remaining five gene structures, there was either no suitable consensus in any frame (one case) or a consensus in an alternative frame that would result in a much smaller protein (four cases) and it does not seem likely that these alternative starts are relevant.

354 of the 387 full-length ORFs terminate in the last exon of the gene structure. A further 27

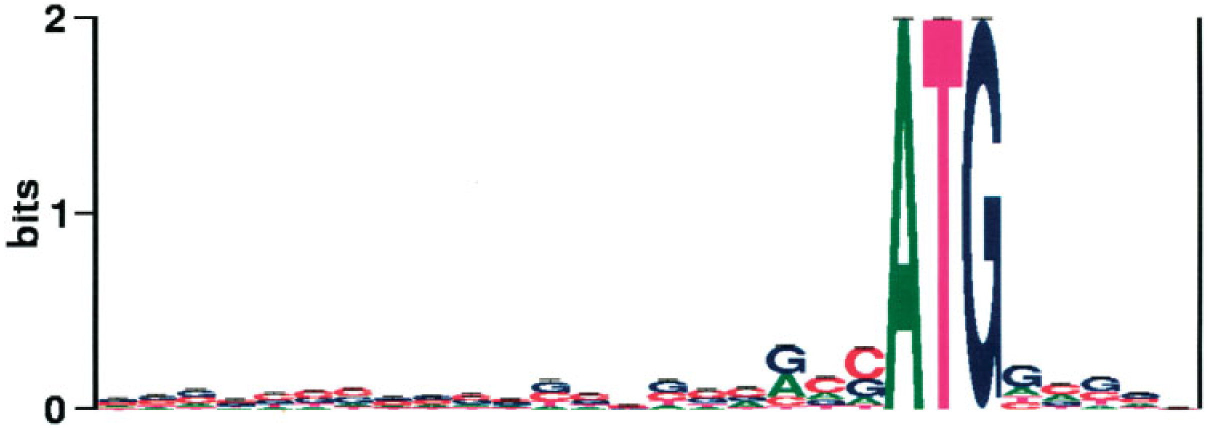

**Figure 9.** Sequence Logo (Schneider and Stephens, 1990) of Kozak site surrounding the initiator ATG

Copyright © 2003 John Wiley & Sons, Ltd. 

ORFs terminate in the last but one exon of the gene, and 19 of these terminate within 50 bases of the last splice junctions. Thus 96.4% of the annotations satisfy the criteria to avoid degradation by nonsense-mediated decay (Maniatis and Reed, 2002; Maquat and Serin, 2001). Of the remaining 14 exceptions, eight ORFs terminate in the last but one exon but are greater than 50 bases from the splice junction. As far as we can tell, these ORFs look bona fide, and either nonsense-mediated decay tolerates these examples or it plays some role in regulation. For the remaining exceptions, it is possible that alternative transcripts to the canonical form that we annotated are the major functional form. At the 5′ end of the ORF there is slightly less restriction as to the exon in which the ORF begins, although the first two exons are still heavily favoured. 64% of ORFs initiate in the first exon, 25% in the second exon, 7.5% in the third exon, 2.5% in the fourth, and the remainder in either the fifth or sixth exons.

### Conclusions

We have outlined the genomic and coding properties of a highly curated set of human protein-coding genes. Notwithstanding the caveat that this set represents one particular region of the genome that may be unusual in terms of its GC content, it seems likely that these properties will be characteristic of most human genes. To further validate this gene set we are now cloning and sequencing cDNAs to obtain a representative clone for each full-length ORF. These clones should be useful not only to verify the collection but also to provide evidence for the existence of each protein through expression studies, generation of antibodies and perhaps RNAi in tissue culture. In this way, we expect to begin to generate an information-rich functional annotation for at least this 1% of the human genome.

### Acknowledgements

This work was supported by the Wellcome Trust. The authors wish to thank all their colleagues who have contributed to the work on chromosome 22 over the years, and Charlotte Cole for critical review of the manuscript.

### References

Bashiardes S, Lovett M. 2000. cDNA detection and analysis. *Curr Op Chem Biol* **5**: 15–20.

Beaudoing E, Freier S, Wyatt JR, *et al.* 2000. Patterns of variant polyadenylation signal usage in human genes. *Genome Res* **10**: 1001–1010.

Chen C, Gentles AJ, Jurka J, Karlin S. 2002. Genes, pseudogenes, and Alu sequence organization across human chromosomes 21 and 22. *Proc Natl Acad Sci USA* **99**: 2930–2935.

Clay O, Caccio S, Zoubak S, *et al.* 1996. Human coding and noncoding DNA: compositional correlations. *Mol Phylogenet Evol* **5**: 2–12.

Collins JE, Goward ME, Cole CG, *et al.* 2003. Reevaluating human gene annotation: a second-generation analysis of chromosome 22. *Genome Res* **13**: 27–36.

Das M, Burge CB, Park E, *et al.* 2001. Assessment of the total number of human transcription units. *Genomics* **77**: 71–78.

Davuluri RV, Grosse I, Zhang MQ. 2001. Computational identification of promoters and first exons in the human genome. *Nature Genet* **29**: 412–417.

Down TA, Hubbard TJ. 2002. Computational detection and location of transcription start sites in mammalian genomic DNA. *Genome Res* **12**: 458–461.

Dunham I, Hunt AR, Collins JE, *et al.* 1999. The DNA sequence of human chromosome 22. *Nature* **402**: 489–495.

Duret L, Mouchiroud D, Gautier C. 1995. Statistical analysis of vertebrate sequences reveals that long genes are scarce in GC-rich isochores. *J Mol Evol* **40**: 308–317.

Ewing B, Green P. 2000. Analysis of expressed sequence tags indicates 35 000 human genes. *Nature Genet* **25**: 232–234.

Flicek P, Keibler E, Hu P, *et al.* 2003. Leveraging the mouse genome for gene prediction in human: from whole-genome shotgun reads to a global synteny map. *Genome Res* **13**: 46–54.

Guigo R, Agarwal P, Abril JF, *et al.* 2000. An assessment of gene prediction accuracy in large DNA sequences. *Genome Res* **10**: 1631–1642.

Guigo R, Dermitzakis ET, Agarwal P, *et al.* 2003. Comparison of mouse and human genomes followed by experimental verification yields an estimated 1019 additional genes. *Proc Natl Acad Sci USA* **100**: 1140–1145.

International Human Genome Sequencing Consortium. 2001. Initial sequencing and analysis of the human genome. *Nature* **409**: 860–921.

Kozak M. 1999. Initiation of translation in prokaryotes and eukaryotes. *Gene* **234**: 187–208.

Liang F, Holt I, Pertea G, *et al.* 2000. Gene index analysis of the human genome estimates approximately 120 000 genes. *Nature Genet* **25**: 239–240.

Maniatis T, Reed R. 2002. An extensive network of coupling among gene expression machines. *Nature* **416**: 499–506.

Maquat LE, Serin G. 2001. Nonsense-mediated mRNA decay: insights into mechanism from the cellular abundance of human Upf1, Upf2, Upf3, and Upf3X proteins. *Cold Spring Harb Symp Quant Biol* **66**: 313–320.

Mittman S, Guo J, Emerick MC, Agnew WS. 1999. Structure and alternative splicing of the gene encoding $\alpha$1I, a human brain T calcium channel $\alpha$1 subunit. *Neurosci Lett* **269**: 121–124.

Roest Crollius H, Jaillon O, Bernot A, *et al.* 2000. Estimate of human gene number provided by genome-wide analysis using Tetraodon nigroviridis DNA sequence. *Nature Genet* **25**: 235–238.

Scherf M, Klingenhoff A, Werner T. 2000. Highly specific localization of promoter regions in large genomic sequences by

Copyright © 2003 John Wiley & Sons, Ltd. 

PromoterInspector: a novel context analysis approach. *J Mol Biol* **297**: 599–606.
Schneider TD, Stephens RM. 1990. Sequence logos: a new way to display consensus sequences. *Nucleic Acids Res* **18**: 6097–6100.
Shoemaker DD, Schadt EE, Armour CD, *et al*. 2001. Experimental annotation of the human genome using microarray technology. *Nature* **409**: 922–927.
Stephens RM, Schneider TD. 1992. Features of spliceosome evolution and function inferred from an analysis of the information at human splice sites. *J Mol Biol* **228**: 1124–1136.
Wright FA, Lemon WJ, Zhao WD, *et al*. 2001. A draft annotation and overview of the human genome. *Genome Biol* **2**: RESEARCH0025.

Copyright © 2003 John Wiley & Sons, Ltd. 
